# The influence of fat substitution with κ‐carrageenan, konjac, and tragacanth on the textural properties of low‐fat sausage

**DOI:** 10.1002/fsn3.620

**Published:** 2018-04-16

**Authors:** Mehdi Atashkar, Mohammad Hojjatoleslamy, Leila Sedaghat Boroujeni

**Affiliations:** ^1^ Department of Food Science and Technology Shahrekord Branch Islamic Azad University Shahrekord Iran; ^2^ Department of Food Science and Technology Science and Research Branch Islamic Azad University Tehran Iran

**Keywords:** κ‐carrageenan, fat substitutes, konjac, sausage, texture, tragacanth

## Abstract

Reducing the fat content of meat products and producing healthier products is considered as an important matter in politics in prevention of many hazardous diseases and providing consumers' health. The aim of this study was reducing the fat in fatty sausages based on oil reduction and using fat substitutes, including κ*‐carrageenan*, konjac, and tragacanth, and comparing them according to their texture characteristics. κ‐carrageenan, konjac, and tragacanth gums were used at four different levels (0.0, 0.5, 1.0, and 1.5) as the fat substitutes in producing low‐fat sausage with 70% reduction based on formulated oil. Texture profile analysis (Hardness, Gumminess, Springiness, and Chewiness) was performed in this study for analyzing the texture characteristics, in 1‐, 10‐, 20‐, and 30‐day time intervals after production. The results showed that producing low‐fat sausage was possible using all the three gums, among the low‐fat samples of which, the texture samples containing konjac were more favorable. Textural properties indicated that fat reduction increased in firmness and gum addition can partially compensate deficits in rheological properties, although during the storage, low‐fat sausages without any gum have highest decline in Hardness. Konjac gum illustrated the best theological properties between treatments.

## 1 INTRODUCTION

Nowadays, the two subjects, safety and nutritional value are resulted to concerns for consumers (Soltanizadeh & Kadivar, 2011). Empirical findings indicate the food fat is the stimulant of various cancer diseases rather than being the staring factor for them (Malek, 2005).

Thus, reducing fat is considered as an important policy for modification of the fat rate and producing healthier product. This is specifically seen about the meat industry, especially since some of the meat products contain extensive rate of fat, and their fat is considered as a dangerous factor for consumers' health (Jiménez‐Colmenero et al., 2012).

Sausage is a uniform mixture resulted from slaughtered livestock, fat, and water filled with other materials in natural or artificial covers in appropriate conditions, prepared for human consumption, after proper heating process and other necessary processes, in such a way that the main materials (meat, fat, and water) should be mixed during the process that they are not separated after heating and have slicing potentials with appropriate consistency (Codex Aimentarius, 2015). Therefore, fat is one of the main elements in producing this food, affecting the organoleptic features of meat products (Cierach, Modzelewska‐Kapituła, & Szaciło, 2009). Producing low‐fat product may cause some technologic problems due to the effects they can have on texture (Cierach et al., 2009). As a lubricating element, fats improve edibility characteristic. Reducing or eliminating it produces a product with dry and elastic texture (Soltanizadeh & Kadivar, 2011). Maintaining appropriate texture characteristics in low‐fat or nonfat food materials is considered a main problem, in this respect. Due to some texturing materials, the required texture by the consumers can be produced in compound food materials (Bourne 2002). This is possible using fat substitutes, such as hydrocolloids, vegetable proteins, and connective tissue proteins (Cierach et al., 2009). The use of carbohydrates, especially hydrocolloids, as the fat substitutes in meat products modifies texture and sensory characteristics of meat products (Farahnaki, Majzoubi, & Mesbahi, 2008).

One of the best and most effective fat substitutes is κ‐carrageenan (Cierach et al., 2009). Using κ‐carrageenan has been common in meat product industry for many years, having appropriate effects, and the effects of other compositions on the products or as the fat substitutes can even be compared with it. The other compound in this regard is konjac (glucomannane) (Jiménez‐Colmenero et al., 2012; Soltanizadeh & Kadivar, 2011). This composition is obtained from the yam roots, a polysaccharide that is classified under glucomannane species (Soltanizadeh & Kadivar, 2011). The main composition of konjac powder is glucomannane, called konjac mannane (Phillips & Williams, 2009). Konjac powder is considered as a safe and low‐calorie compound containing indigestible fibers and has various physiologic effects with therapeutic applications (González Canga et al., 2004). The other composition is tragacanth, which is among the secretory gums and one of the first emulsifiers and stabilizers, used in little amounts in food materials (Alemzadeh, Amin Mohammadifar, Azizi, & Ghanati, 2010; Farahnaki et al., 2008). The suitable oral sense and texture can be created with appropriate amounts of tragacanth gum. The aim of this study was producing three types of low‐fat sausage according to reducing formulated oil and using fat substitutes, including κ‐carrageenan, konjac, and tragacanth hydrocolloids, and comparing the effects of each one on Hardness, Gumminess, Springiness, and Chewiness of low‐fat sausage texture characteristics and also comparing them with each other.

## 2 MATERIALS AND METHODS

### 2.1 Formulation

The amount of beef fat (the purchased beefsteak from Mataboi Co., Brazil) was balanced with the rate of 12% for formulation and producing the sausage sample with 40% of red meat. According to Table 1, the raw materials, including oil (Naz Co., Iran), flour (Joree Co., Iran), gluten and starch (Shahdineh Aran Co., Iran), salt (Goharnab Co., Iran), poly phosphate sodium (Budenheime Co., Germany), ascorbic acid and sodium nitrite (Basf Co., Germany), garlic and spices (local market), ice and water (local market), κ‐carrageenan gels with 10% (sifted with sieve no. 120 from CEAMSA Co., Spain), konjac flour (sifted with sieve no. 120 from Gemfont Co., Taiwan), and tragacanth (produced from Astragalus gossypinus Fischer, milled and sifted with sieve no. 120 purchased from the local market) were crushed and mixed in Mini‐cutter (Minika, Spain) for about 10 min, under temperature conditions of under 6°C, and the resulted mixture was packed in a 60‐mm diameter polyamide cover by the filling device (Handtmann Inc., Germany). Then, the sausages went under 72–75°C for 100 min by water steam in the curing compartment, and immediately after that, the temperature was reduced to less than 30°C by cold air (0–4°C). The products were then quarantined and kept in the cold storage with 0–4°C, for the required tests (Rokni, 1998).

**Table 1:** Formulation of produced sausage samples

No.	Raw materials	Control sample (Fatty)	Samples without gum	Samples containing 0.5% gum	Samples containing 1% gum	Samples containing 1.5% gum
1	Red meat(with 12% fat)	40.00	40.00	40.00	40.00	40.00
2	Water and ice	26.31	37.86	37.36	36.86	36.36
3	Oil	16.50	4.95	4.95	4.95	4.95
4	Flour	6.00	6.00	6.00	6.00	6.00
5	Gluten	4.00	4.00	4.00	4.00	4.00
6	Starch	3.00	3.00	3.00	3.00	3.00
7	Spices	1.40	1.40	1.40	1.40	1.40
8	Salt	1.40	1.40	1.40	1.40	1.40
9	Garlic	1.00	1.00	1.00	1.00	1.00
10	Sodium phosphate	0.35	0.35	0.35	0.35	0.35
11	Ascorbic acid	0.03	0.03	0.03	0.03	0.03
12	Sodium nitrite	0.01	0.01	0.01	0.01	0.01
13	Gum^a^	0.00	0.00	0.50	1.00	1.50

a It is to note that depending on the type of samples, k‐carrageenan, konjac, and tragacanth were used separately in the formulation.

### 2.2 Texture profile analysis

Texture profile analysis (TPA) was used to evaluate texture characteristics, in 1‐, 10‐, 20‐, and 30‐day time intervals after production. Brookfield texture analysis (Model: CT3‐4500, Brookfield engineering, USA) at a constant crosshead velocity 1 mm/s, Trigger point of 4.5 g, and cylindrical sausage samples with the diameter of about 23 mm and height of about 20 mm were used for TPA evaluation.

Each test was performed with at least three repetitions. Hardness, Springiness, Gumminess, and Chewiness factors were evaluated in the test (Bourne, 2002; Jiménez‐Colmenero et al., 2010). Gumminess and Chewiness are functions of Hardness and cohesiveness, in addition as described in Equation (2) Chewiness in also dependent on Springiness.

(1) Gumminess=Hardness.Cohesiveness

(2) $$\begin{array}{cc} \text{Chewiness}= & \text{Hardness}.\text{Cohesiveness}.\text{Springiness}\\ & =\text{Gumminess}.\text{Springiness} \end{array}$$

### 2.3 Statistical analysis

One‐way ANOVA was used to determine statistical difference between treatments (*p* < .05), for this purpose SPSS ver 19 has been used.

(Valizadeh & Moghadam, 2010).

## 3 RESULTS

According to Table 2 for the comparison of mean values, it can be found that reducing oil and fat substitutes with κ‐carrageenan reduces the rate of Hardness as compared to the control samples, such that this rate has significant reduction in the samples with no gum, but by increasing the gum (0.5% level), this rate gets significant increase relative to the samples without gum (*p* < .05), and by concentration increase(1% level), the Hardness gets a higher increase, and at 1.5% level of the gum, the Hardness reaches to less than the 1% level.

**Table 2:** The effects of different percentages of replacing k‐carrageenan, konjac, and tragacanth on Hardness of low‐fat sausage samples (*p* < .05)

Treatment	Day 1	Day 10	Day 20	Day 30
Control sample	4174.17 ± 292.83^eD^	1412.50 ± 230.55^hA^	3396.83 ± 54.62^hB^	3842.33 ± 67.28^gC^
Sample with no gum (0%)	9322.33 ± 188.19^bD^	4227.00 ± 48.03^bB^	5223.00 ± 68.54^bC^	2170.08 ± 21.67^bA^
Sample with 0.5% k‐carrageenan	6309.33 ± 136.71^dC^	2980.60 ± 54.90^deB^	9260.17 ± 30.87^dD^	2572.33 ± 118.80^dA^
Sample with 1% k‐carrageenan	1321.33 ± 182.64^dA^	1318.17 ± 65.12^fA^	6306.33 ± 82.77^fC^	3025.83 ± 89.05^efB^
Sample with 1.5% k‐carrageenan	3316.67 ± 151.74^dC^	2316.17 ± 78.46^efA^	3304.67 ± 124.04^efAC^	2915.67 ± 81.49^eB^
Sample with 0.5% konjac	9305.33 ± 114.70^dC^	6285.43 ± 116.23^cdB^	2537.89 ± 61.54^dA^	2319.33 ± 5.48^cA^
Sample with 1% konjac	1332.83 ± 148.01^dA^	1311.43 ± 79.13^efA^	2910.33 ± 80.61^eB^	2661.83 ± 70.01 ^dB^
Sample with 1.5% konjac	8399.67 ± 108.75^eC^	4356.00 ± 75.45^gB^	3317.50 ± 47.80^gA^	3090.17 ± 68.57^fA^
Sample with 0.5% tragacanth	3272.00 ± 72.44^cB^	8268.00 ± 107.92^cC^	2505.67 ± 105.60^cdA^	2401.33 ± 89.86^cA^
Sample with 1% tragacanth	2240.67 ± 28.65^bA^	6238.83 ± 66.01^bB^	2384.50 ± 103.52^cA^	2383.17 ± 51.43^cA^
Sample with 1.5% tragacanth	3199.17 ± 4.65^aB^	2198.17 ± 68.54^aA^	1978.17 ± 91.78^aA^	1963.17 ± 68.57^aA^

Different uppercase letters indicate significant differences (*p*<0.05) between treatments for the same time. Different lowercase letters indicate significant differences (*p*<0‐05) between times for the same treatment.

According to Table 3 for the comparison of mean values, it can be found that reducing oil and fat substitute with κ‐carrageenan reduces the Gumminess as compared to control samples, such that the Gumminess of the samples without gum has significant reduction as compared to the control samples (*p* < .05), but it increases significantly by adding the κ‐carrageenan in 0.5 and 1% levels. Then, by increasing the substitute level in 1.5% level, the Gumminess decreases to lower level than 1% of gum, and this rate shows a significant reduction in all levels as compared to the control samples (*p* < .05). Thus, adding κ‐carrageenan in all levels increases the Gumminess in low‐fat sausages, the reason of which is that as Gumminess is the result of the product of the two Hardness and cohesiveness factors in each other, then due to increasing the Hardness in the sausage because of adding the gum, the required energy is also increased for disjoining the sausage for swallowing (Ayadi, Kechaou, Makni, & Attia, 2009; Hellyer, 2004). Moreover, after 30 days of preserving time, Gumminess shows reduction in all samples, which is only significant for the samples containing 0.5% κ‐carrageenan (*p* < .05).

**Table 3:** The effects of different percentages of replacing k‐carrageenan, konjac, and tragacanth on Gumminess of low‐fat sausage samples (*p* < .05)

Treatment	Day 1	Day 10	Day 20	Day 30
Control sample	7289.70 ± 239.22^gC^	1289.45 ± 207.63^fA^	5287.40 ± 33.98^gB^	8279.32 ± 46.07^gD^
Sample with no gum (0%)	5166.44 ± 37.58^bcC^	1166.13 ± 53.59^bcA^	2165.54 ± 75.94^bcB^	7162.87 ± 35.57^bD^
Sample with 0.5% k‐carrageenan	8196.73 ± 32.13^deD^	5196.50 ± 69.98^deC^	4172.45 ± 65.67^bcdB^	3172.37 ± 73.83^bcA^
Sample with 1% k‐carrageenan	9211.67 ± 61.56^eC^	7210.85 ± 60.13^eB^	2070.00 ± 64.98^fA^	9200.98 ± 82.80^fC^
Sample with 1.5% k‐carrageenan	9196.00 ± 32.07^deC^	8196.44 ± 45.67^deB^	4194.50 ± 82.57^efA^	9193.55 ± 95.04^efC^
Sample with 0.5% konjac	2030.40 ± 117.58^eB^	4196.30 ± 73.79^deC^	7177.14 ± 71.96^cdD^	1700.84 ± 49.90^bcA^
Sample with 1% konjac	3213.87 ± 149.71^eC^	2040.73 ± 70.63^eA^	2194.74 ± 118.56^efB^	5180.60 ± 69.67^cdD^
Sample with 1.5% konjac	3244.70 ± 51.33^fC^	4213.75 ± 72.98^eD^	1950.00 ± 46.48^efA^	2181.10 ± 83.61^cdeB^
Sample with 0.5% tragacanth	7178.30 ± 53.02^cdD^	3180.33 ± 85.87^cdB^	4185.50 ± 102.21^deC^	2188.70 ± 114.27^defA^
Sample with 1% tragacanth	1510.20 ± 18.93^bA^	3153.50 ± 126.57^bB^	5161.60 ± 64.74^bC^	5163.50 ± 43.13^bC^
Sample with 1.5% tragacanth	895.87 ± 108.54^aB^	496.32 ± 86.31^aA^	1010.67 ± 33.98^aB^	3102.40 ± 62.52^aC^

Different lowercase letters indicate significant differences (*p*<0.05) between treatments for the same time. Different uppercase letters indicate significant differences (*p*<0‐05) between times for the same treatment.

According to Table 4 (comparison of mean values), it can be found that reducing oil and fat substitute with κ‐carrageenan reduces the Springiness as compared to control samples, the Springiness of the samples without gum has little reduction as compared to the control samples, but it increases partially by increasing the gum in 0.5% level. Then, the gum obtains a higher Springiness by increasing the gum concentration in 1% level, and at 1.5% level, the Springiness reduces to lower than 1% level, but no significant difference is observed between the samples (*p* > .05). Therefore, adding κ‐carrageenan increases the Springiness partially in low‐fat sausage, which is in conformity with the results obtained by Pietrasik and Jarmoluk (2003) about the obtained gel from port. Moreover, after 30 days of preserving time, Springiness shows a partial increase in all samples (*p* > .05).

**Table 4:** The effects of different percentages of replacing k‐carrageenan, konjac, and tragacanth on Springiness and Chewiness of low‐fat sausage samples (*p* < .05)

Treatment	Day 1	Day 10	Day 20	Day 30
Control sample	7.11 ± 0.11^cA^	7.15 ± 0.11^bA^	7.19 ± 0.26^bA^	7.21 ± 0.09^cdA^
Sample with no gum (0%)	6.78 ± 0.37^bcA^	6.97 ± 0.15^bA^	7.03 ± 0.14^bA^	7.09 ± 0.10^bcdA^
Sample with 0.5% k‐carrageenan	6.79 ± 0.22^bcA^	6.85 ± 0.76^bA^	6.87 ± 0.20^bA^	6.90 ± 0.07^bA^
Sample with 1% k‐carrageenan	7.04 ± 0.12^cA^	7.05 ± 0.26^bA^	7.10 ± 0.15^bA^	7.12 ± 0.2^bcdA^
Sample with 1.5% k‐carrageenan	6.82 ± 0.35^bcA^	6.86 ± 0.22^bA^	6.88 ± 0.21^bA^	6.92 ± 0.12^bcA^
Sample with 0.5% konjac	6.97 ± 0.22^bcA^	7.00 ± 0.31^bA^	7.12 ± 0.14^bA^	7.13 ± 0.25^bcdA^
Sample with 1% konjac	7.06 ± 0.20^cA^	7.09 ± 0.18^bA^	7.21 ± 0.32^bA^	7.24 ± 0.32^dA^
Sample with 1.5% konjac	6.56 ± 0.20^abA^	6.76 ± 0.36^abA^	6.93 ± 0.13^bA^	6.97 ± 0.04^bcA^
Sample with 0.5% tragacanth	6.96 ± 0.90^bcA^	7.13 ± 0.55^bAB^	7.15 ± 0.11^bB^	7.18 ± 0.09^bcdB^
Sample with 1% tragacanth	6.86 ± 0.24^bcA^	6.98 ± 0.32^bA^	6.91 ± 0.21^bA^	6.93 ± 0.01^bcA^
Sample with 1.5% tragacanth	6.33 ± 0.25^aA^	6.35 ± 0.62^aA^	6.39 ± 0.13^aA^	6.41 ± 0.09^aA^

Different lowercase letters indicate significant differences (*p*<0.05) between treatments for the same time. Different uppercase letters indicate significant differences (*p*<0‐05) between times for the same treatment.

As shown in Table 4, it can be found that reducing oil and fat substitute with konjac reduces the Springiness as compared to control samples, the Springiness of the samples without gum has a partial reduction as compared to the control samples (*p* > .05), but it increases partially by increasing the gum in 0.5% level (*p* > .05). Then, the gum obtains a higher Springiness by increasing the density of the gum in 1% level, and at 1.5% level, the Springiness reduces to lower than 1% level (*p* < .05). Therefore, adding konjac in 0.5% and 1% levels partially increases the Springiness in low‐fat sausages. Moreover, after 30 days of preserving time, Springiness shows a partial increase in all the samples.

As expressed in Table 5, oil and fat substitution with κ‐carrageenan reduced Chewiness compared with control samples, such that the Chewiness of the samples without gum has significant reduction as compared to the control sample (*p* < .05), and by adding gum in 0.5% level as a partial increase, a 1% level significant increase (*p* > .05) is shown as compared to the control sample and also in the 1.5% level of the gum it shows a partial increase (*p* > .05), and a significant reduction is seen in all levels compared with the control sample (*p* < .05), compared to the samples without gum (*p* < .05), but it increases partially by increasing the tragacanth with 0.5% level (*p* > .05), and by increasing the substitute level, it has a higher reduction (*p* > .05), such that the reduction in 1.5% level of gum is significant as compared to the samples without gum (*p* < .05). Overall, the rate of Springiness of all the samples showed a reduction as compared to the control sample. Therefore, increasing tragacanth in 0.5% and 1% levels partially increases Chewiness in low‐fat sausages. Moreover, after 30 days of preserving time, Chewiness shows a partial increase in all the samples, which is significant only for the samples containing 0.5% tragacanth (*p* < .05).

**Table 5:** The effects of different percentages of replacing k‐carrageenan, konjac, and tragacanth on Chewiness of low‐fat sausage samples (*p* < .05)

Treatment	Day 1	Day 10	Day 20	Day 30
Control sample	197.53^fA^ (24.13)	196.96^dA^ (4.11)	196.23^eA^ (8.02)	195.32^eA^ (6.80)
Sample with no gum (0%)	113.41^bcA^ (4.90)	112.93^bcA^ (5.60)	112.28^bcA^ (9.83)	111.71^bcA^ (1.00)
Sample with 0.5% k‐carrageenan	131.94^cdeA^ (10.11)	129.16^bcA^ (10.56)	115.96^bcA^ (5.47)	114.91^bcA^ (10.66)
Sample with 1% k‐carrageenan	146.40^deA^ (4.34)	145.63^cA^ (28.49)	143.84^dA^ (20.68)	140.36^dA^ (19.07)
Sample with 1.5% k‐carrageenan	131.70^cdA^ (7.34)	131.54^bcA^ (21.10)	131.43^bcdA^ (4.00)	131.37^bcdA^ (10.58)
Sample with 0.5% konjac	140.83^deA^ (9.28)	135.83^bcA^ (10.15)	126.65^bcdA^ (15.79)	121.25^bcdA^ (7.34)
Sample with 1% konjac	147.26^deA^ (12.74)	141.73^cA^ (22.58)	136.13^cdA^ (18.60)	127.94^bcdA^ (17.28)
Sample with 1.5% konjac	153.11^eA^ (14.63)	143.84^cA^ (15.32)	137.57^cdA^ (15.57)	128.26^bcdA^ (10.47)
Sample with 0.5% tragacanth	126.96^cdA^ (7.39)	128.34^bcA^ (34.76)	132.29^bcdA^ (19.84)	135.80^cdA^ (25.21)
Sample with 1% tragacanth	100.68^bA^ (6.29)	104.64^bA^ (17.71)	108.80^bA^ (7.97)	109.86^bA^ (7.94)
Sample with 1.5% tragacanth	59.70^aA^ (8.98)	60.95^aA^ (13.94)	62.77^aA^ (9.29)	63.00^aA^ (6.48)

Different lowercase letters indicate significant differences (*p*<0.05) between treatments for the same time. Different uppercase letters indicate significant differences (*p*<0‐05) between times for the same treatment.

## 4 DISCUSSION

As illustrated in Table 2, Hardness showed a significant decrease (in different treatments) compared with the control samples (*p* < .05). Candogan and Kolsarici (2003), Matulis, McKeith, Sutherland, and Brewer (1995), He and Sebranek (1996) also believe that by increasing the density of κ‐carrageenan, the texture of low‐fat frankfurters becomes harder. The results have had conformity up to 1% level in this study. Moreover, according to the viewpoint of Ayadi et al. (2009), the reason for increasing the Hardness up to 1% level of the gum can be due to the presence of additional κ‐carrageenan in the formed gel network. Thus, as expected, adding of κ‐carrageenan in all levels improves (increases) Hardness in low‐fat sausage, and according to the viewpoint of Verbeken, Neirinck, Van Der Meeren, and Dewettinck (2005), one of its reasons can be increasing the Hardness of the obtained gel from soluble proteins in meat salt, and this result is in conformity with the results obtained by Pietrasik and Jarmoluk (2003), Xiong, Noel, and Moody (1999), Ruusunen et al. (2003), and also García‐García and Totosaus (2008). Furthermore, after 30 days of storage, the rate of Hardness showed reduction in all the samples, which was significant only for the samples containing 0.5% and 1.5% levels of κ‐carrageenan (*p* < .05). The Hardness reduction during the time of keeping was in conformity with the results obtained by Ayadi et al. (2009), about the effect of κ‐carrageenan on turkey sausage.

According to Table 2, for the comparison of mean values and, it can be found that reducing oil and substitutes of fat with konjac reduce the Hardness as compared to the control samples, such that the Hardness of the samples without gum has significant reduction as compared to the control samples (*p* < .05), but by adding the gum (0.5% level), the Hardness shows significant reduction as compared to the samples without gum, and by increasing the substitute level (1% and 1.5% levels), the Hardness shows a higher increase (*p* < .05). The rate of Hardness of the samples containing gum shows a significant reduction as compared to the control samples (except 1.5% level with a negligible reduction) (*p* < .05). Thus, adding konjac in all levels increases Hardness in low‐fat sausage, which is in conformity with the results obtained by Liaros, Katsanidis, and Bloukas (2009) and also Ruiz‐Capillas, Triki, Herrero, Rodriguez‐Salas, and Jiménez‐Colmenero (2012), one reason of which is that due to heating of konjac gel, hydrogen bonds are weakened, and hydrophobic mutual reactions (polymer–polymer) are improved, and Hardness increases by stabilization of konjac gel (Hellyer, 2004). Moreover, after 30 days of preserving, Hardness shows reduction in all samples, which is significant for the samples containing konjac (*p* < .05). According to Triki, Herrero, Jiménez‐Colmenero, and Ruiz‐Capillas (2013), one of the reasons of softening of the texture in low‐fat samples as compared to control samples is due to increasing the rate of humidity to the rate of protein. According to Table 2 for the comparison of mean values, it can be found that reducing oil and fat substitute with tragacanth reduces the Hardness as compared to control samples, such that the Hardness of the samples without gum has significant reduction as compared to the control samples, but it increases significantly by adding the gum (*p* < .05). Then, by increasing the substitute level, the Hardness decreases (*p* < .05). Thus, adding tragacanth in 0.5% and 1% levels increases the Hardness in low‐fat sausages. Moreover, after 30 days of shelf life, Hardness shows reduction in all samples, which is only significant for the samples containing 0.5% tragacanth (*p* < .05). Generally, the texture is softened in low‐fat samples as compared to control samples, which is in conformity with the results obtained by Claus, Hunt, and Kastner (1989), Hensley and Hand (1995), and also Candogan and Kolsarici (2003), the reason of which is probably according to the viewpoint of Triki et al. (2013) due to increasing the humidity ratio to protein. Also, according to Ruusunen et al. (2003), when fat is replaced with water in equal weights in the formulation, the texture becomes softer, Sutton, Hand, and Newkirk (1995) stated that there is a negative correlation between Hardness and humidity in frankfurters. Generally, regarding Hardness, the highest rate of it was related to the control samples, and the lowest rate of it was related to the samples containing tragacanth with 1.5% level, which showed significant changes (*p* < .05). According to Table 3 for the comparison of mean values, it can be found that reducing oil and fat substitute with konjac reduces the Gumminess as compared to control samples, such that by adding gum (in 0.5% and 1% levels), the Gumminess of the samples shows significant increase (*p* < .05), and by increasing the substitute level in 1.5% level of gum, the Gumminess shows again a significant increase as compared to the samples without gum and shows significant reduction as compared to the control samples in all levels (*p* < .05) decreases to lower level than 1% of gum, and this rate shows a significant reduction in all levels as compared to the control samples (*p* < .05). Thus, adding konjac in all levels improves Gumminess in low‐fat sausage, and the reason for the increase is that since Gumminess is the result of the product of the two Hardness and cohesiveness factors in each other. According to the mean comparison Table 3, it can be realized that reducing oil and fat substitute with tragacanth decreases Gumminess as compared to the control samples, such that it has significant reduction in the samples without the gum as compared to the control samples (*p* < .05), and by adding tragacanth (in 0.5% level), the amount of it has a partial increase as compared to the samples without the gum (*p* > .05), and by increasing the substitute level (1% and 1.5% levels), in comparison with the samples without gum, the rates show a partial reduction (*p* > .05) and significant reduction, respectively, indicating significant reduction in all levels as compared to the control samples (*p* < .05). Therefore, adding tragacanth has caused increasing Gumminess in low‐fat sausage, only in 0.5% level, and the reason for the increase is due to increasing Hardness of the sausage because of adding the gum (only in this level, as compared to the samples without gum), and the required energy is increased for disjoining the sausage for swallowing (Ayadi et al., 2009; Hellyer, 2004). Moreover, after 30 days of preserving time, Gumminess shows a partial reduction in control samples and samples with no gum, but it shows a partial increase in the samples containing tragacanth (*p* > .05).

Generally, regarding Gumminess, the highest rate of it is related to the control samples, and the lowest rate is related to the sample containing tragacanth with 1.5% level, and the changes are significant (*p* < .05). Moreover, the closest samples compared with the control samples are the ones containing 1.5% konjac (*p* > .05).

Table 4 showed, fat reduction using tragacanth led to reduction in Springiness, such that the Springiness of the samples without gum has a partial reduction as compared to the control samples, but it increases partially by increasing the tragacanth with 0.5% level (*p* > .05), and by increasing the substitute level, it has a higher reduction (*p* > .05), such that the reduction in 1.5% level of gum is significant as compared to the samples without gum (*p* < .05). Overall, the rate of Springiness of all the samples showed a reduction as compared to the control samples. Therefore, increasing tragacanth in 0.5% and 1% levels partially increases Springiness in low‐fat sausages. Moreover, after 30 days of preserving time, Springiness shows a partial increase in all the samples, which is significant only for the samples containing 0.5% tragacanth (*p* < .05).

Generally, regarding Springiness, the highest rate of it is related to the control sample, and the lowest rate is related to the sample containing tragacanth with 1.5% level, and the changes are significant (*p* < .05). Moreover, the closest samples compared with the control sample are the ones containing 1% konjac, and no significant difference is observed between the two samples (*p* > .05).

The increasing rate of Chewiness up to 1% of the gum is in conformity with the results obtained from Ayadi et al. (2009), about the effect of κ‐carrageenan on turkey sausage. Thus, adding κ‐carrageenan in all levels improves the Chewiness in low‐fat sausages, which is in conformity with the research by Pietrasik and Jarmoluk (2003) about the results on pork, and the reason for the increase is that since Chewiness is the result of the product of the two Gumminess and Springiness factors in each other, then according to the increase in Gumminess and Springiness of sausage because of adding gum as compared to the samples without the gum, the required energy for oral digestion of sausage for swallowing is increased (Ayadi et al., 2009; Hellyer, 2004). Moreover, after 30 days of preserving time, Chewiness shows a partial decrease in all the samples (*p* > .05). According to Table 5 (comparison of mean values), it can be found that reducing oil and fat substitute with konjac reduces the Chewiness as compared to control samples, such that the Chewiness of the samples without gum has significant reduction as compared to the control samples (*p* < .05), and by adding gum and increasing the substitute level, Chewiness has found a rather significant increase as compared to the samples without the gum (*p* < .05), which is in conformity with results obtained by Ruiz‐Capillas et al. (2012); that is, by reducing fat and increasing the gum level, the required energy for chewing has a significant increase, but it has shown a significant reduction in all levels as compared to the control samples (*p* < .05). Thus, adding konjac in all levels improves Chewiness in low‐fat sausage, and it is in conformity with the results obtained by Ruiz‐Capillas et al. (2012), and the reason for the increase is that since Chewiness is the result of the product of the two Gumminess and Springiness factors in each other, then according to the increase in Gumminess and Springiness of sausage (except 1.5% level) because of adding gum as compared to the samples without the gum, the required energy for oral digestion of sausage for swallowing is increased (Ayadi et al., 2009; Hellyer, 2004). Moreover, after 30 days of preserving time, Chewiness shows a partial decrease in all the samples (*p* > .05).

According to Table 5 (comparison of mean values), it can be found that reducing oil and fat substitute with tragacanth reduces the Chewiness as compared to control samples, such that the Chewiness of the samples without gum has significant reduction as compared to the control samples (*p* < .05), but by adding gum (at 0.5% level), Gumminess increases a little (*p* > .05), and by increasing the substitute level (1% and 1.5% levels), the Chewiness is reduced to the extent that its rate as compared to the samples without gum shows partial reduction (*p* > .05) and significant reduction (*p* < .05), respectively, having significant reduction in all levels as compared to the control sample (*p* < .05). Therefore, adding tragacanth in only 0.5% level partially increases Chewiness in low‐fat sausages, and the reason for the increase is due to increasing Gumminess and Springiness of the sausage because of adding the gum (only in this level, as compared to the samples without gum), and the required energy is increased for oral digestion of the sausage for swallowing (Ayadi et al., 2009; Hellyer, 2004). Moreover, after 30 days of preserving time, Chewiness shows a partial reduction in control samples and samples with no gum, but it shows a partial increase in the samples containing tragacanth (*p* > .05).

Generally, regarding Chewiness, the highest rate of it is related to the control sample, and the lowest rate is related to the samples containing tragacanth with 1.5% level, and the changes are significant (*p* < .05). Moreover, the closest samples compared with the control sample are the ones containing 1.5% konjac (*p* > .05).

## 5 CONCLUSION

Comparison between important factors that may effective on the customer acceptance, textural factors have the most important position. As shown, considering the textural factors such as Hardness, Gumminess, Springiness, and Chewiness, sample containing 1.5% konjac is more favorable to formulate low‐fat sausage than k‐carrageenan and tragacanth.

## CONFLICT OF INTEREST

The authors declare no conflict of interest.
